# A Prognostic Gene Signature for Metastasis-Free Survival of Triple Negative Breast Cancer Patients

**DOI:** 10.1371/journal.pone.0082125

**Published:** 2013-12-11

**Authors:** UnJin Lee, Casey Frankenberger, Jieun Yun, Elena Bevilacqua, Carlos Caldas, Suet-Feung Chin, Oscar M. Rueda, John Reinitz, Marsha Rich Rosner

**Affiliations:** 1 Ben May Department for Cancer Research, University of Chicago, Chicago, Illinois, United States of America; 2 Department of Oncology, University of Cambridge, Cambridge, United Kingdom; 3 Departments of Statistics, Ecology and Evolution, Molecular Genetics and Cell Biology, University of Chicago, Chicago, Illinois, United States of America; Queen's University Belfast, United Kingdom

## Abstract

Although triple negative breast cancers (TNBC) are the most aggressive subtype of breast cancer, they currently lack targeted therapies. Because this classification still includes a heterogeneous collection of tumors, new tools to classify TNBCs are urgently required in order to improve our prognostic capability for high risk patients and predict response to therapy. We previously defined a gene expression signature, RKIP Pathway Metastasis Signature (RPMS), based upon a metastasis-suppressive signaling pathway initiated by Raf Kinase Inhibitory Protein (RKIP). We have now generated a new BACH1 Pathway Metastasis gene signature (BPMS) that utilizes targets of the metastasis regulator BACH1. Specifically, we substituted experimentally validated target genes to generate a new BACH1 metagene, developed an approach to optimize patient tumor stratification, and reduced the number of signature genes to 30. The BPMS significantly and selectively stratified metastasis-free survival in basal-like and, in particular, TNBC patients. In addition, the BPMS further stratified patients identified as having a good or poor prognosis by other signatures including the Mammaprint® and Oncotype® clinical tests. The BPMS is thus complementary to existing signatures and is a prognostic tool for high risk ER-HER2- patients. We also demonstrate the potential clinical applicability of the BPMS as a single sample predictor. Together, these results reveal the potential of this pathway-based BPMS gene signature to identify high risk TNBC patients that can respond effectively to targeted therapy, and highlight BPMS genes as novel drug targets for therapeutic development.

## Introduction

The application of gene expression array technology to breast cancer has emphasized the heterogeneity of this disease and also provided new tools to classify breast cancers into subtypes based on gene expression patterns. Ideally each subtype would reflect distinct molecular characteristics corresponding to discrete cancer phenotypes. This information could be used to gain prognostic insight and, eventually, to predict response to therapy. In addition to the traditional clinical parameters (size, grade and node status) and pathological markers (ER, PR and HER2 status), breast cancer can be classified into at least 5 ‘intrinsic’ subtypes (Luminal A, Luminal B, HER2-enriched, Basal-like, Normal-like) that were derived from a hierarchical clustering analysis of expression profiles of human breast tumors [1], [2]. This classification has generated a gene-expression predictor, the PAM50 Classifier, that measures the expression of 50 genes to establish the intrinsic tumor subtypes and has been useful as a prognostic marker but has not yet reached its potential impact on clinical care [3].

Recently other gene expression signatures have been developed in order to stratify patients by survival and to provide more accurate prognostic tools [4]–[10]. Most of these signatures however identify a few groups of patients that are mainly separated based on ER status, HER2 status and proliferation markers and thus partially overlap with the molecular subtyping [4]. Supervised analysis of expression data has also led to clinical assays like the OncotypeDX®, a diagnostic test that analyzes expression of 21 genes and provides a likelihood of recurrence for early stage, estrogen receptor positive (ER+) patients [5]. Similarly, Mammaprint® analyzes the expression of 70 genes, mostly related to proliferation, and can stratify early-stage, node negative patients based on the risk of recurrence [6]. Both these tests have a prognostic significance but their applicability with respect to targeted therapy is primarily limited to a well defined group of patients whose tumors express ER or HER2.

One of the main challenges in the breast cancer field is to gain a better knowledge of the biology of triple negative (ER-/PR-/HER2-) breast cancer (TNBC) in order to develop clinical approaches to this disease. TNBC represents 14 to 20 percent of all breast cancers, has a high incidence in young women, is more frequent in African American women compared to Caucasian, and is often associated with BRCA1 mutations [11]. TNBC represents the most aggressive type of breast cancer and the one with the poorest prognosis. This is due in part to the fact that there is no targeted therapy available and in part because of the high risk of recurrence. Moreover, recurrence occurs generally within a few years and often involves metastasis, especially to the brain and lung. TNBC largely comprises a subset of basal-like breast tumors. Although chemotherapy is often initially beneficial in basal-like tumors, those with residual disease after treatment have a high risk of relapse [12]. Targeted therapy has potential value for treatment; however, it is important to first identify the subpopulations of patients that are most at risk.

We previously defined a signaling pathway-based gene signature named the *RKIP* pathway metastasis signature (RPMS) that is predictive for metastasis-free survival in a heterogeneous cohort of breast cancer patients [13]–[15]. This signature was based upon statistically determined regulatory relationships that were experimentally validated and then applied using a cut-off based model. These include the metastasis suppressor gene Raf Kinase Inhibitory protein (*RKIP*), targets of the downstream let-7 microRNA family including the pro-metastatic *let-7* targets BACH1 and *HMGA2*, and finally their downstream targets *MMP1*, *CXCR4* and *OPN*. We experimentally demonstrated that the microRNA let-7 suppresses breast cancer metastasis, and BACH1, a leucine zipper transcription factor, promotes breast cancer metastasis. By basing prognostic signatures for TNBC patient survival on signaling pathway information, it is theoretically possible to identify drug targets that will enable effective response of this patient cohort to treatment.

Our present goal is to improve the RPMS to make it more clinically relevant and more targeted to specifically discriminate among subgroups of TNBC patients. We used new gene expression array data, obtained using a TNBC cell line, to experimentally define the BACH1 target genes. Using this refined set of genes, we then applied an optimization process to gene expression data from human breast tumors to obtain a prognostic signature. Finally, we added the capability of being a single sample predictor. We thus define a novel BACH1 pathway metastasis signature (BPMS) and show that it functions as a prognostic indicator of metastasis-free survival in a heterogeneous cohort of patients as well as TNBC patients. In addition, because the BPMS is based on a signaling pathway, it also has the potential for guiding the development of new therapy targeted to genes within this signaling network that promote metastasis in TNBC patients.

## Materials and Methods

### Breast Cancer Patient Tumor Datasets

Three datasets on the Affymetrix hg-u133a platform were assembled and utilized: BrCa871 (n = 871) and BrCa443 (n = 443), and BrCa341 (n = 341). The training set BrCa871, consisting of 871 patients, contains five cohorts identified by their GEO accession numbers: GSE1456, GSE2990, GSE3494, GSE7390, and GSE11121. The first testing data set BrCa443 (443 patients) is composed of three cohorts: GSE5327, GSE2034, and GSE2603 and the second testing set BrCa341 (341 patients) is also composed of three cohorts: GSE6532, GSE12093, GSE31519. These datasets were RMA pre-processed, median centered by sample, and z-score transformed. One further dataset, METABRIC [16], was also utilized for validation. For details on dataset composition as well as preprocessing methodology, see Methods S1. The BrCa871 set was split into two sets for training purposes: BrCa436-Train for training and BrCa435-CV for cross-validation. BrCa341 and BrCa443 were not utilized in the training process and used only for validation. A further dataset consisting of genes regulated by shBACH1 depletion in breast tumor cells was generated to identify potential genes of interest but was not used for training purposes. All data was analyzed using R.

### Generation of stable cell lines, RNA isolation and microarray analysis

Stable depletion of *BACH1* in MDA-MB-231-derived 1833 (also termed BM1) human metastatic breast cancer cells was achieved using shRNA lentiviral vectors as described previously [15]. RNA was isolated from cells using RNeasy Mini Kit according to manufacturer's instruction (Qiagen) and reverse transcription was performed as described previously [11]. Affymetrix GeneChip Human Gene 1.0 ST arrays were used for expression analysis of RNA samples, in triplicate, from 1833 cells expressing shBACH1 or a scrambled control RNA (3x scrambled RNA control and 3x shBACH1 accessible as GSE50226). All microarray data (including both cell line and patient tumor gene expression data) were preprocessed using the Robust Multi-array Average (RMA) framework (R Bioconductor libraries ‘oligo’ and ‘pd.hugene.1.0.st.v1′); samples were then median-centered by subtracting the median expression value from each sample.

### Generation of let-7-TG and BACH1 meta-genes

A high-confidence set of *let-7* target genes was previously generated using target prediction programs [14]. A list of *BACH1* target genes was generated by analyzing differences in expression levels between control and sh*BACH1* 1833 cells using the Significance Analysis in Microarrays package (R library ‘samr’) with a high stringency cutoff (median FDR = 0.125; p<0.001) [17]. The lists of both significantly up-regulated and down-regulated genes were imported into DAVID for annotation of global function-related themes [18].

Meta-genes were constructed as previously described [14], [18] (see Methods S1 for detailed description). Briefly, downstream targets of *let*-7 and *BACH1* were combined into weighted averages to serve as an estimate of regulation by both the microRNA (*let*-7) and the transcription factor (*BACH1*). As *let-7* suppresses its downstream targets, an increase in *let-7* should cause a decrease in the overall *let-7* target gene meta-gene (meta-let-7-TG). Conversely, as *BACH1* activates its downstream targets, an increase in *BACH1* should cause a net increase in the *BACH1* target gene meta-gene (meta-BACH1). The meta-genes serve to define activity of these regulators in individual patients relative to *RKIP* expression.

### Threshold selection and Cost Function Optimization Overview

In order to find a set of cutoff values for the genes in the signature that was significant and also remained prognostic across multiple datasets, we treated the problem as an inversion and optimization problem. A cost function was formulated to reflect significance using the logrank test p-value and well as cohort size. All p-values are logrank p-values unless otherwise noted. Furthermore, all survival data was right-censored at 5 years with the exception of the training sets. Cutoff values were adjusted to minimize the cost function using a non-linear optimizer. In this case, we utilized the Nelder-Mead algorithm natively in R (R function ‘optim’) to find local minima of the cost function.

We utilized the BrCa871 dataset as the overall training set and the BrCa443 and BrCa341 datasets as the testing sets. An additional dataset, the METABRIC, was also used as a validation set [16]. It is important to note that the BrCa443 and BrCa341 datasets are independent datasets and were never utilized in the entire training process. Furthermore, the cell line data was not utilized for training or validation but only for gene selection. The BrCa871 dataset was separated into two smaller sets of approximately the same size: a training set including 436 patients (BrCa436-Train) and a cross-validation set of 435 patients (BrCa435-CV). A series of 24,800 potential combinations of cutoffs was first generated in the BrCa436-Train set by minimizing the cost function. Of these, 556 combinations produced a significant P-value in both BrCa436-Train and BrCa435-CV and for each gene the mean was calculated from these 556 significant cutoffs, yielding the final set of cutoff values.

### Signaling System Model

In our system, we are primarily concerned with the consequences of *RKIP* suppression. Using relationships between genes previously demonstrated [12], we hypothesize that *RKIP* suppression should reduce expression of *let-7*. Since *let-7* inhibits *BACH1* and *HMGA2*, suppression of *let-7* should activate both *BACH1* and *HMGA2*. Similarly, activation of both *BACH1* and *HMGA2* should induce activation of *MMP1*, *CXCR4*, and *OPN*. Summation over the *d* function (equation 2) returns values between 0 and 7. Values less than 7 represent incomplete pathway activation, while values exactly equal to 7 indicate that the entire BPMS pathway is activated. Given information on either complete or incomplete activation, our classifier function (equation 1) returns a value of either 0 or 1. If the sample's gene expression values are consistent with complete RKIP pathway activation, the function's output is 1. Otherwise, if at least one gene in the RKIP pathway does not properly reflect complete activation, the function's output is 0.

The BACH1 Pathway Metastasis Signature functions as a classifier between high risk and low risk of future metastasis when applied to a 2-dimensional data matrix 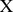 of gene expression values with elements 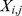 representing the 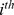
gene of the 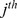
sample. Our classifier function, designed to reflect pathway activation in downstream targets of RKIP and BACH1, is written,
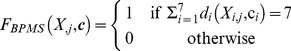
(1)

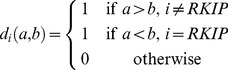
(2)


Here, 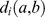 is the thresholding/activation function for gene, and 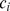 is our corresponding threshold. These thresholds were trained on gene expression values running from 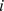  = 1 through 7, representing *RKIP*, meta-let-7-TG, meta-BACH1, *HMGA2*, *MMP1*, *CXCR4*, and *OPN* respectively. With the exception of meta-let-7-TG, if a given gene's expression levels are greater than its threshold, that gene is said to be activated; similarly, if the same gene's expression levels are less than its threshold, it is said to be repressed. Since the meta-let-7-TG is an aggregation of various downstream targets of let-7, suppression of let-7 should cause an overall increase in meta-let-7-TG. Therefore, if meta-let-7-TG is greater than its threshold, let-7-TG is said to be activated. Inherent to this methodology is an inverse relationship between the number of gene-parameters and the predicted size of cohorts identified.

### Cost Function

The aim of the classifier function is to demonstrate within a specified subpopulation of breast cancer patients a correlation between the mRNA expression values of our given set of BPMS genes and the phenotype of decreased likelihood of metastasis-free survival. Therefore, we sought a relationship between the relative expression levels of our 7 BPMS genes and certain statistical properties of the BPMS subpopulation. In order to increase the predictability and effectiveness of the classifier function, we searched for a set of thresholds that simultaneously maximizes the size of the potential BPMS subpopulation, and minimizes the metastasis-free survival stratification of that subpopulation. To that end, we defined a cost function whose parameters are the expression levels of the 7 genes and whose values are a linear sum of functions α and β. α (equation 4) is a discretization of the raw log-rank p-value of the potential BPMS cohort reflecting the significance of the potential solution; β (equation 5) is a linear transformation of the relative proportion of BPMS patients, reflecting the effect size (patient population size) of the solution. By optimizing α, we select for solutions that maximize the significance of the signature. However, to avoid over-fitting for significance, optimizing the β function selects for solutions that maximize the effect size of the signature.

An alternative statistical parameter to the log-rank p-value that can be directly interrogated by the function α is the hazard ratio. However, the hazard ratio can be shown to be simply a linear transformation of raw log-rank values. Raw log-rank values go to a chi-squared distribution, and we are using the p-values on the extreme end of the chi-squared distribution. Therefore, the hazard ratio (to a high approximation) can be explained linearly as a function of the log-rank p-value.

We minimized the cost function using a numerical optimizer. We now describe these steps in detail.

The cost function's first component α was discretized to match the discrete nature of the second component β. Similarly, the range of β for typical parameter values was roughly scaled to match the range of α. The net effect of this discretization and scaling is the creation of a very frugal cost function that rejects small changes that merely add a patient or two and instead rewards larger jumps that drastically change the raw log-rank p-value of potential BPMS thresholds.

Our cost function 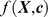 is written as,
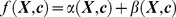
(3)where
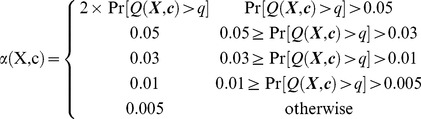
(4)and
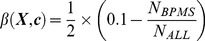
(5)



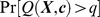
is the log-rank p-value of our potential BPMS cohort, 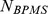
is the number of potential BPMS patients, and 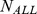
is the total number of patients in 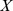.

### Optimizer

The optimizer used to produce solutions was the default R implementation (R function optim) of the downhill simplex algorithm [19]. Random solutions drawn from a normal distribution with mean zero and variance one were selected as starting points.

### Survival analysis

To determine the significance of differential survival between BPMS and non-BPMS patients, the logrank test was performed on annotated metastasis-free survival (MFS) data paired to each sample. Kaplan-Meier plots were also generated for each dataset to provide a visualization of survival stratification. Comparisons of five year survival were determined using right-censoring of survival data in all validation sets.

When assessing the overall significance of the BPMS compared to other prognostic signatures, an analysis of variance (ANOVA) test was performed. To compare two models in an ANOVA, a hypothesis test called the likelihood ratio test can be performed. The likelihood ratio test compares the ratio of likelihoods for a given multivariate Cox model (e.g. published prognostic signatures) relative to a second model (e.g the published prognostic signatures plus the BPMS) and determines whether or not a particular regressor (e.g. BPMS) imparts significant information to the first model. The ANOVA utilizes repeated application of the likelihood ratio test from a null model to a full model by successively adding single prognostic signatures. Thus, using multivariate Cox proportional hazards models, we calculated the significance of the BPMS when compared to all other prognostic signatures examined. For more information on the multivariate ANOVA test, see Methods S1.

### BPMS on Alternate Array Platforms

To test the BPMS for cross platform compatibility, we utilized the METABRIC expression array dataset of 2000 breast tumors performed on the Illumina BeadArrays [16]. To compare survival we applied the BPMS as above in these datasets and compared metastasis-free survival using the logrank test on Kaplan-Meier survival curves.

### Signature Comparisons

Implementation of intrinsic subtype, proliferation, triple-negative, Mammaprint®, Oncotype®, GAB2 signaling scaffold, 28-kinase metagene, glucocorticoid receptor, and 76-gene signatures were performed as previously described [1], [2], [4]–[8], [10], [20], [21]. Comparisons to intrinsic subtyping, proliferation, GAB2 signaling scaffold, 28-kinase metagene, glucocorticoid receptor and triple-negative signatures were used to demonstrate the significance of the BPMS within basal breast cancer populations. Further comparisons to the 76-gene signature were used to predict overall survival, and Mammaprint and Oncotype signatures were included to establish the complementarity of the BPMS signature to these clinically-relevant signatures. Patient subgroup survival was compared in a pairwise manner using the logrank test (R library ‘survival’) and across the combination of all signatures using the multivariate likelihood ratio/ANOVA test.

### Software Code

All of the code used to perform the analyses is included in the supplementary documents.

## Results

### Analysis of gene expression changes in a BACH1-depleted TNBC cell line and generation of meta-genes

To build a BACH1 pathway metastasis signature (BPMS), we determined whether the use of experimentally derived BACH1 targets to build the *BACH1*-metagene could reduce the number of genes included in the signature and improve the ability of our signature to predict patient outcome. Meta-genes combine the individual expression of a group of genes into a single value. For the RPMS signature [13]–[15], we used meta-genes as surrogates for *let-7* and *BACH1* since expression of their target genes could reflect their activity better than their expression level alone. Moreover, the meta-gene was necessary for estimating the level of *let-7* expression, as its expression level was not measured directly on the Affymetrix hgu133a platform. The *BACH1* meta-gene was built in order to estimate the level of transcriptionally active BACH1 as its activity is regulated at multiple levels including cofactor association and cytoplasmic sequestration [22], [23]. The original *BACH1* meta-gene in the RPMS was based on predicted targets for BACH1 obtained from the TRANSAFAC database [14].

To build a new BACH1 meta-gene, we stably depleted *BACH1* via shRNA transfection of 1833 cells, a bone tropic derivative of MDA-MD-231 TNBC cells [24]. We performed microarray analysis on these cells and identified a group of genes that had significant (p<0.001) differential expression following *BACH1* depletion. Specifically, 80 genes were increased and 88 genes were decreased (Table S1). Using the functional annotation software tool DAVID, we determined that *BACH1* expression correlated positively with genes in categories related to the cytoskeleton and extracellular matrix including actin-binding to Wiskott-Aldrich homology 2 (WH2), extracellular/secreted and EGF-like. BACH1 expression correlated negatively with genes in categories related to phospholipid metabolism including calcium binding, sterile alpha motif, inositol phosphate metabolism, plasma membrane and phospholipase activity. These results are consistent with previous findings demonstrating that BACH1 promotes breast cancer metastasis [14], [25].

We utilized the experimentally-derived BACH1 target genes to minimize the number of components required to generate meta-genes in order to facilitate clinical application of the new BACH1-based signature. The RPMS signature was comprised of approximately 100 genes of which most were contained in the *BACH1* TG meta-gene. To reduce the number of genes that act as surrogates of *BACH1*, we determined the variance of each gene across 871 gene expression arrays conducted on resected breast cancer tumors (termed the BrCa871 training dataset) and filtered the BACH1 targets by selecting genes with the lowest variance. A similar analysis was conducted for *let-7* TG. This procedure yielded a list of 12 genes for the *let-7* TG meta-gene and 13 genes for the *BACH1* meta-gene (Table 1). The new *let-7* meta-gene is a subgroup of the one we used previously [14]. By contrast, the new *BACH1* meta-gene has no genes in common with those in the RPMS and thus had to be further tested to assess how well it represents BACH1 as a component of the RKIP signaling pathway.

**Table 1:** Gene targets comprising the *let*-7 meta-gene (left) and BACH1 meta-gene (right).

Let-7 Targets		BACH1 Targets	
**ARID3B**	AT rich interactive domain 3B (BRIGHT-like)	**BMPER**	BMP binding endothelial regulator
**CCNJ**	cyclin J	**DYM**	dymeclin
**GOLT1B**	golgi transport 1B	**FBXO42**	F-box protein 42
**HIC2**	hypermethylated in cancer 2	**FRMPD4**	FERM and PDZ domain containing 4
**IGF2BP3**	insulin-like growth factor 2 mRNA binding protein 3	**HERC3**	HECT and RLD domain containing E3 ubiquitin protein ligase 3
**IL13**	interleukin 13	**HS3ST3B1**	heparan sulfate (glucosamine) 3-O-sulfotransferase 3B1
**MAP4K4**	mitogen-activated protein kinase kinase kinase kinase 4	**IL1RAP**	interleukin 1 receptor accessory protein
**NF2**	neurofibromin 2 (merlin)	**IL7**	interleukin 7
**PAPPA**	pregnancy-associated plasma protein A, pappalysin 1	**MAGEC1**	melanoma antigen family C, 1
**SLC6A1**	solute carrier family 6 (neurotransmitter transporter, GABA), member 1	**MYCT1**	myc target 1
**TGFBR1**	transforming growth factor, beta receptor 1	**PDE1C**	phosphodiesterase 1C, calmodulin-dependent 70 kDa
**ZC3H3**	zinc finger CCCH-type containing 3	**PRDM1**	PR domain containing 1, with ZNF domain
		**RCAN3**	RCAN family member 3

### *let-7-TG* and *BACH1* meta-genes correlate to other components of the RKIP signaling pathway and to previous meta-genes

In order to test if the new *let-7-TG* and *BACH1* meta-genes behave as elements of the RKIP signaling cascade and maintain the expected correlation to other components of the pathway in patient datasets, we performed gene set analysis [13]. As observed previously, expression of the genes that we selected as *let-7* targets (meta-let-7-TG) correlated inversely to *RKIP* expression when tested as a set (p<0.001, FDR<0.001, score = −0.46). Similarly, expression of the BACH1 target set (metaBACH1) correlated positively to the *let-7-TG* meta-gene, to *BACH1* expression and to the BMS gene set when tested using the BrCa871 dataset (scores = 1.06, 0.93, and 1.60, respectively; p<0.001, FDR<0.001 for all). To determine whether these newly defined meta-genes for *let-7* TG and BACH1 are representative of the *let-7-TG* and *BACH1* meta-genes used for the RPMS, we correlated the distributions of these two sets of meta-genes across the BrCa871 dataset. Analysis yielded a Pearson correlation of 0.71 for the *let-7-TG* meta-genes and 0.69 for *BACH1* meta-genes. These results showed a high degree of correlation between the respective meta-genes suggesting that the newly created meta-genes are a good representation of the old one when interrogated using breast cancer patient gene expression data.

We then determined whether these new meta-genes follow a normal distribution. Initially, the preprocessing approach and the Central Limit Theorem ensured that all genes in all datasets that we used in this study were distributed normally. However, it is possible that our processing method for creating meta-genes engendered major bias. To test this possibility, we generated Q-Q plots for the *let-7-TG* and *BACH1* meta-genes. The results indicate that each meta-gene is extremely linear in this representation and thus is normally distributed (Figure S1).

### Setting signature cutoffs using cost function optimization and cross validation

Although our previous RPMS gene signature used a median cutoff for individual genes to stratify patients, the median cutoff is an arbitrary value and may not be the most appropriate way to establish gene activity. The median cutoff assumes that the threshold for activation is the same for all genes and corresponds to the median value. To improve threshold selection beyond the median cutoff, we developed a novel methodology involving cost function optimization.

Specifically, we utilized a mathematical approach that optimized the cost function to define the most effective gene cutoffs for stratifying patients while maximizing the patient group size. To accomplish this, we used the R implementation of Nelder-Mead optimization (R function ‘optim’), setting the cutoffs of the genes and meta-genes in the signature (5 genes and 2 meta-genes) as the values to be optimized. Using a subset of the BrCa871 dataset (BrCa436-Train), we optimized our cost function by adjusting the cutoffs, thereby maximizing power and specificity. Instead of using the median value as a cutoff for all genes, we chose a value for each gene that was able to maximize significant differences in metastasis-free survival.

**Figure 1:**
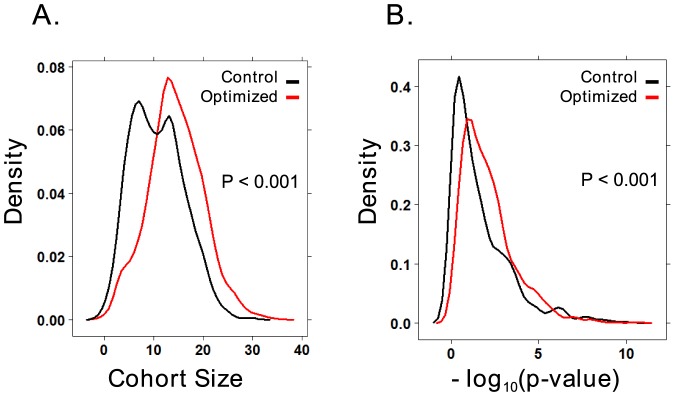
The optimized solutions yield larger cohort sizes and better p-values. Distribution density plots for non-optimized (control) and optimized signatures verify that significantly better cohort sizes (A) and p-values (B) were generated using a cost function in conjunction with the Nelder-Mead optimization algorithm.

As a control to determine whether the optimizer will yield better solutions than non-optimized solutions, the cost function was optimized 1444 times using the BrCa871 dataset. We compared these optimized results to a set of 1056 randomly generated solutions that were not optimized. We then analyzed the p-values (statistical significance) and cohort size (number of patients expressing the 7 gene signature) of these solutions. The optimized solutions yielded an average p-value of 0.0868 with a variance of 0.0041 while also yielding an average cohort size of 14.43 with a variance of 0.15. The random solutions yielded a mean p-value of 0.223 with a variance of 0.008 as well as an average cohort size of 10.82 with a variance of 0.16. Using a t-test to compare the two, we found that the optimized solutions give significantly better p-values (p<0.0001), as well as a significantly larger cohort size (p<0.0001) (Figure 1A, B). These results indicate that optimization over the cost function produces significantly better thresholds for gene expression than random methods.

**Figure 2:**
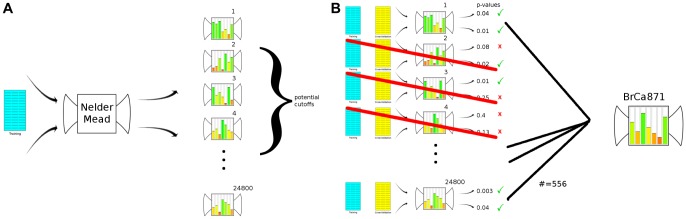
Optimization procedure for the BPMS. After separating the overall training set (BrCa871) into a training set and a cross validation set, (A) a series of 24,800 potential solutions are produced by optimizing our cost function using the Nelder-Mead downhill simplex algorithm. These solutions were trained on survival data with no year-specific endpoint defined to maximize signal sensitivity (See Figure 4). Using these 24,800 potential solutions, (B) significance in both training and cross-validation sets was assessed. To control for over-fitting solutions, 556 solutions yielding significance in both sets were extracted and used to estimate the final BPMS signature.

To build a predictive model, results were obtained from 24800 optimizations in BrCa436-Train and cross-validated using the remainder of the data (BrCa435-CV) as a control for over-fitting. Specifically all 24800 potential solutions were applied to the BrCa435-CV dataset. Solutions that did not produce significance in both the training as well as cross-validation sets were discarded, leaving a remaining 556 potential solutions. Using this underlying distribution of significant solutions (p<0.05), an estimate of the final set of the cutoffs was generated (Figure 2). The final cutoff values were set by averaging the results within each gene (or meta-gene) using normalized (0, 1) data that was median-centered by patient. This analysis generated 7 cutoff values: −0.27 for *RKIP*, −0.23 for *MMP1*, 0.19 for *OPN*, −0.20 *HMGA2*, −0.19 for *CXCR4*, −0.020 for meta-let7-TG, and −0.15 for meta-BACH1. These results identified a new metastasis gene signature with a greatly reduced number of genes. We term this new signature the BACH1 pathway metastasis signature or BPMS.

### BPMS as a single sample predictor

Ideally, for clinical purposes, a prognostic signature would enable one to predict survival for a single sample independent of the context of a larger patient population. Since the previous RPMS gene signature used a median cutoff for individual genes that cannot be defined outside of a statistical distribution, the RPMS cannot be applied on a patient-to-patient basis. Individual patients may be added to already existing distributions of gene expression values, but the addition of each patient would, in fact, change the median threshold for each gene. Similarly, using RMA preprocessing as above prevents us from generating a single sample predictor (SSP) from the BPMS. An alternative approach is to use the frozen RMA (fRMA) package (Bioconductor package ‘fRMA’) [21] to perform quantile normalization and pre-processing of all samples. Unlike RMA, which calculates normalization parameters using a given dataset, fRMA utilizes a “frozen” set of parameters that are independent of other samples within a dataset.

**Figure 3:**
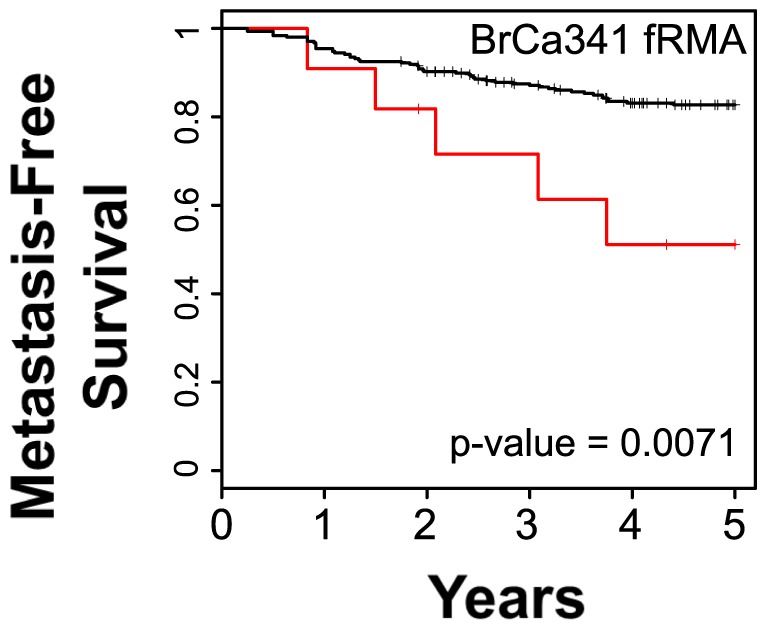
The BPMS is a single patient predictor. Using frozen RMA pre-processed data, the BPMS was trained to be applied on a patient-to-patient basis. The BrCa871 set was processed using fRMA, divided into the BrCa436-Train and BrCa435-CV sets and 7,500 potential solutions were optimized. Using a cross-validation strategy, a final set of BPMS parameters were trained for fRMA processed data. Shown is the application of these parameters to the fRMA processed BrCa341 data set.

To determine whether the BPMS can function as a single sample predictor (SSP), the BrCa871 and BrCa341 datasets were processed using fRMA. After splitting BrCa871 into both BrCa436-Train and BrCa435-CV, 7500 solutions were trained on BrCa436-Train. These 7500 solutions were then cross-validated using BrCa435-CV, and solutions that did not produce significance within both BrCa436-Train and BrCa435-CV were discarded. All remaining solutions were averaged to yield a single sample predictor. This SSP version of the BPMS was then validated using fRMA-processed BrCa341 data (Figure 3). The results indicate that the BPMS, when used as a SSP, has the potential to significantly predict patient survival.

### Signature hypothesis testing

To address concerns that random gene signatures of a similar size are equally effective or even more significant at stratifying patient data than our experimentally derived BPMS, we used a Monte Carlo method to sample 1,520 sets of 7 random genes [26]. We ran optimizations over our 1,520 gene sets using identical methodology to that used for analysis of the BPMS target genes. To be specific, we optimized each gene set using the cutoff model on the BrCa436-Train data, selected solutions that produced significance in the BrCa435-CV data, and estimated the most effective cutoff values of the gene set. We then applied the resulting signature for each random gene set to the BrCa443 data, yielding a log-rank p-value for each gene set. We used these 1,520 gene sets to provide an estimate of the proportion of 7-gene permutations that our BPMS gene set outperformed. The BPMS out-performed a significant portion of the randomly produced signatures, yielding a p-value of 0.0389. These results indicate that the group of genes we chose for the signature is significantly different from a random group.

### BPMS is Prognostic for Metastasis-Free Survival

**Figure 4:**
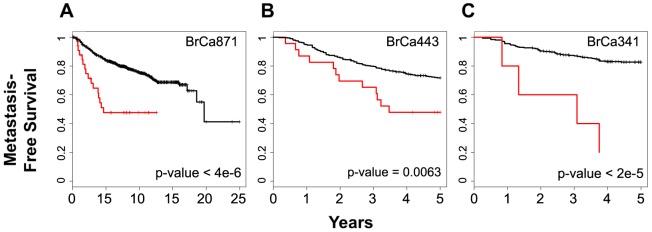
The BPMS is prognostic for metastasis-free survival (MFS). Patients from three breast cancer datasets, (**A**) BrCa871 (35 BPMS+ out of 871 patients), (**B**) BrCa443 (24 BPMS+ out of 443 patients) and (**C**) BrCa341 (6 BPMS+ out of 341 patients), were stratified for MFS using the BPMS. BrCa871 is shown with no year-specific clinical endpoint to reflect the training data. Red indicates patient tumors that express the BPMS signature while black indicates patient tumors that do not. Survival curves were generated by Kaplan–Meier analysis, and the indicated P-values were calculated by the log-rank test.

To determine whether the BPMS is associated with metastatic risk, we performed logrank tests on different breast cancer patient datasets, applying the cutoffs we generated previously. When applied to the entire BrCa871 set that was used for training, the analysis yielded a p-value of less than 4.0×10^−6^. Similarly, analysis of two other datasets, BrCa443 and BrCa341, yielded p-values of 6.3×10^−3^, and <2.0×10^−5^ for 5 year survival, respectively (Figure 4A–C). While a relatively low number of BPMS patients in the BrCa341 set may suggest an instability in the signature, a chi-squared test demonstrates that there is no significant deviation from the expected number of patients when compared to the BrCa871 dataset (χ2 = 3.1657, dof = 1, p = 0.0752). These analyses indicate that the BPMS signature is significant and has prognostic value, effectively stratifying patients for risk of metastasis.

### BPMS stratifies patients identified by other signatures

**Figure 5:**
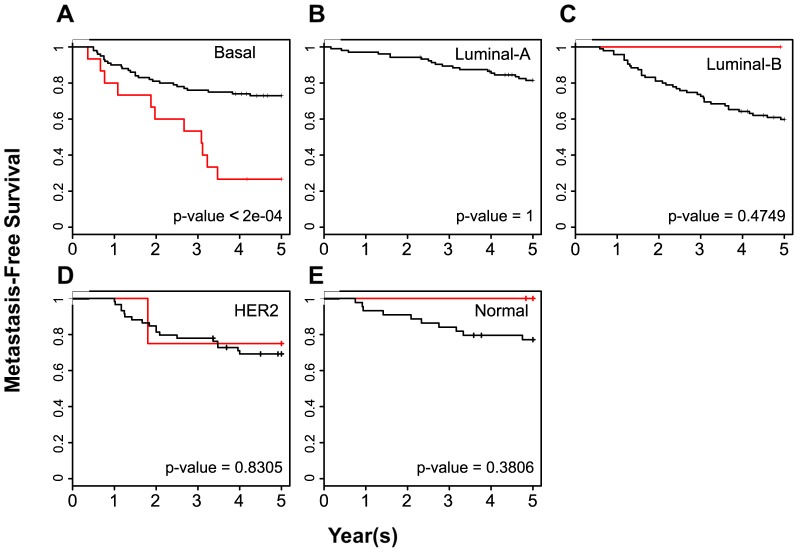
The BPMS is prognostic for metastasis-free survival of breast cancer patients with tumors of the basal subtype. PAM50 was used to categorize breast tumors into (**A**) Basal (16 BPMS+ patients out of 120 Basal patients, χ^2^ = 13.7), (**B**) luminal A (0 BPMS+ patients out of 110 luminal A patients), (**C**) luminal B (1 BPMS+ patient out of 97 luminal B patients, χ^2^ = 0.5), (**D**) HER2 (4 BPMS+ patients out of 67 HER2 patients, χ^2^ = 0) and (**E**) normal (3 BPMS+ patients out of 48 Normal patients, χ^2^ = 0.8) subtypes as indicated. BrCa443 patients were stratified for MFS using the BPMS. Red indicates patient tumors that express the BPMS signature while black indicates patient tumors that do not. Survival curves were generated by Kaplan–Meier analysis, and the indicated P-values were calculated by the log-rank test.

Previous signatures have been used to classify breast cancer patient tumors into molecularly defined groups based on gene expression levels. In addition, these gene signatures have been applied as clinical tests for patient prognosis. Based on these criteria, we defined two categories of signatures, molecular and clinical, and then tested the prognostic value of BPMS patients using the BrCa443 dataset with these two types of signatures. Within the molecular classifiers, the PAM50 signature identifies five subgroups: Luminal A, Luminal B, Normal, Her2+ and Basal. The BPMS patients overlap primarily with basal patients. However, the BPMS significantly enhances patient stratification for MFS (p-value<2×10^−4^; Figure 5A–E) Specifically, the BPMS can significantly differentiate between higher and lower risk patients within the highly aggressive basal subtype.

**Figure 6:**
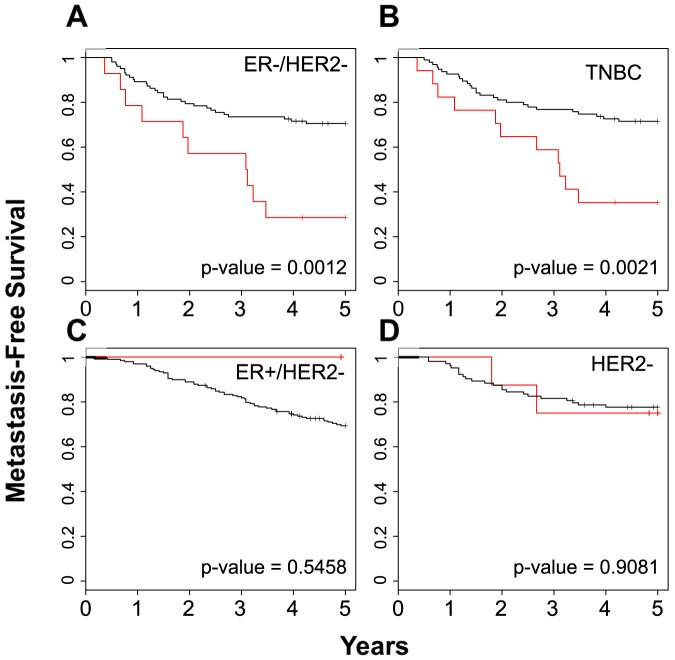
The BPMS is prognostic for metastasis-free survival of TNBC patients. The proliferation signature was used to categorize breast tumors into (**A**) ER-HER2- (15 BPMS+ patients out of 121 ER-HER- patients, χ^2^ = 10.5), (**B**) TNBC (18 BPMS+ patients out of 118 TNBC patients, χ^2^ = 9.4), (**C**) ER+HER2- (n = 1), and (**D**) HER2+ (8 BPMS+ patients out of 117 HER2 patients, χ^2^ = 0). BrCa443 patients were stratified for MFS using the BPMS. Red indicates patient tumors that express the BPMS signature while black indicates patient tumors that do not. Survival curves were generated by Kaplan–Meier analysis, and the indicated P-values were calculated by the log-rank test.

**Figure 7:**
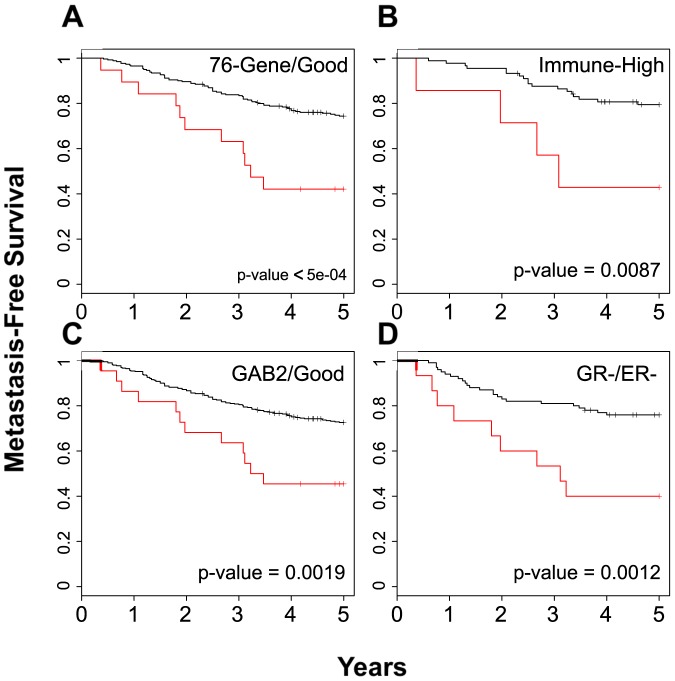
The BPMS is prognostic for high risk patients among good prognosis patients. Good prognosis categories examined were: (**A**) the 76-gene (20 BPMS+ patients out of 290 good-prognosis patients, χ^2^ = 12.2), (**B**) 28-kinase metagene (8 BPMS+ patients out of 104 high immune response patients, χ^2^ = 6.9), (**C**) GAB2 Scaffolding (23 BPMS+ patients out of 429 good prognosis patients, χ^2^ = 9.7), and (**D**) glucocorticoid receptor signature (16 BPMS+ patients out of 121 GR-/ER- patients as defined by 50% cutoff, χ^2^ = 10.5). Patients were stratified for MFS using the BPMS. Red indicates patient tumors that express the BPMS signature while black indicates patient tumors that do not. Survival curves were generated by Kaplan–Meier analysis, and the indicated P-values were calculated by the log-rank test.

Within the molecular phenotypes, we first looked at the proliferation signature, a classification that builds meta-genes to predict whether patients are ER positive or negative as well as Her2 positive or negative. Our analysis indicated that the BPMS patients overlap the ER-/Her2- group, and the BPMS again significantly stratifies them further for MFS (p-value = 0.0012; Figure 6A–C). In addition, TNBC patients, although typically defined through histological assays, were recently categorized by a gene expression signature [18]. TNBC patients identified by this signature significantly overlapped with the BPMS patients; however, as above, the BPMS signature further stratified these patients for MFS (p-value = 0.00214; Figure 6D). Using the entire BrCa443 dataset, we also applied the BPMS to four other molecular signatures: 1) a 76-gene signature predictive of distant metastasis-free survival [7]; 2) a 28-kinase metagene signature related to immune response of cytotoxic T-cells [8]; 3) a 205 gene transcriptional GAB2 scaffold signature related to proliferation and cell adhesion/migration/invasion [9]; and 4) a glucocorticoid receptor signature whose stratification is dependent on ER status [10]. In the latter case, we also analyzed only the top and bottom 25% of the patients as in the original report [10] (Figure S2). Interestingly, in all four cases, the BPMS identified poor prognosis patients within the good prognosis cohort (Figure 7). These results suggest that the BPMS can be used in conjunction with other molecular signatures to identify patients that might otherwise be considered low risk.

**Figure 8:**
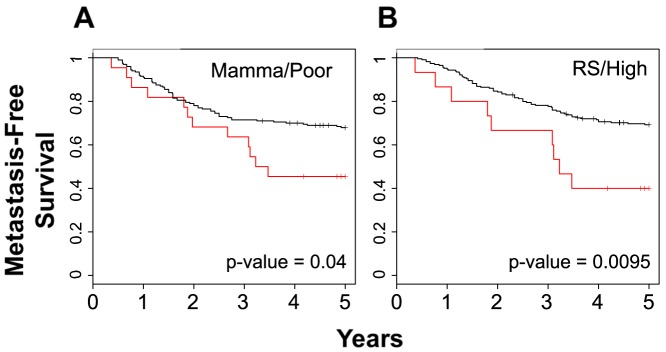
The BPMS is prognostic for high risk patients among the clinically predicted poor outcome and high recurrence patients. Clinically relevant gene signatures (A) Mammaprint® Poor (23 BPMS+ patients out of 226 Mammaprint Poor patients, χ^2^ = 4.3) and (B) OncotypeDX® Recurrence High (16 BPMS+ patients out of 257 RS High patients, χ^2^ = 6.7) were stratified for MFS using the BPMS. Red indicates patient tumors that express the BPMS signature while black indicates patient tumors that do not. Survival curves were generated by Kaplan–Meier analysis, and the indicated P-values were calculated by the log-rank test.

Finally, we applied the BPMS to gene signatures that are currently used in the clinic (OncotypeDX® and MammaPrint®). The BPMS was able to further stratify patients in the poor prognosis subgroup of patients analyzed by Mammaprint® and the high recurrence subgroup of patients analyzed by OncotypeDX® (p-value = 0.04 and 0.01 respectively; Figure 8A,B; Figure S3A-C). Thus, the BPMS gene signature is significantly different than these other signatures and adds information when combined pairwise.

A multivariate analysis of common clinical factors consisting of nodal status, grade, size, ER status, and age was also performed. As clinical data was sparsely available, a combined set of BrCa443 and BrCa341 was used for this analysis. Using the methodology of Sabatier, *et. al.* [8], we first fit univariate Cox models to each clinical factor individually. Of those factors, only nodal status and size were significant predictors on their own. We then fit a multivariate Cox model to nodal status, size, and the BPMS (Table 2). Analysis of variance using a likelihood ratio test with competitive linear Cox models shows that the BPMS significantly adds value independent of clinical factors (p = 0.0073, Survival∼clinical factors vs Survival∼clinical factors+BPMS) (Table 2). Additionally, a similar comparison of all the molecular and prognostic signatures mentioned above indicates that the BPMS signature significantly adds prognostic value to the combined signatures (p = 0.028; Survival∼combined signatures vs. Survival∼combined signatures+BPMS) (Table 3). Together, these analyses show that the BPMS is a significant predictive variable even after adjustment for all available clinical and prognostic factors.

**Table 2:** The BPMS is a significant predictor of metastasis-free survival (MFS) after adjustment for clinical variables.

Table 2A				
	Univariate Analysis		Multivariate Analysis	
Risk Factor	HR (95% CI)	p-value	HR (95% CI)	p-value
Nodal Status	1.47 (1.104–1.971)	0.0086	1.450 (1.0828–1.9425)	0.013
Grade (1, 2 vs 3)	0.872 (0.965–1.029)	0.36		
Size (> vs ≤20 mm)	0.982 (0.9832–0.9915)	0.00017	0.982 (0.9726–0.9907)	0.000083
ER status	1.03 (0.7757–1.358)	0.86		
Age	1 (0.9925–1.015)	0.54		
BPMS	2.3 (1.406–3.762)	0.0009	2.183 (1.3057–3.6495)	0.0029

Table 2B	
Model	Log-likelihood
S∼node+size	−1472.7
S∼node+size+BPMS	−1469.1
−2*(L0 – L1)	7.2
	p-value = 0.0073

= 0.0073, χ^2^ = 7.2, df = 1). All available clinical data in a combined BrCa443/BrCa341 dataset was fit individually to Cox proportional hazards models. A) Clinical factors that were significant univariate predictors of MFS were placed into a full model along with the BPMS. B) An analysis of the variance (likelihood ratio test) comparing the multivariate model with and without the BPMS (L0 and L1 respectively) demonstrates the prognostic ability of the BPMS (p

**Table 3:** The BPMS is a significant predictor of metastasis-free survival (MFS) after adjustment for 7 other prognostic gene signatures.

Table 3A				
Gene Signatures	Hazard Ratio (95% CI)	p-value	Hazard Ratio (95% CI)	p-value
Proliferation Meta-gene: ER+/HER2- vs ER-/HER2-	0.65055 (0.1894–2.2341)	0.4946	0.62060 (0.17661–2.1808)	0.4569
Proliferation Meta-gene: HER2+ vs ER-/HER2-	0.30734 (0.1024–0.9226)	0.0354	0.28245 (0.09221–0.8652)	0.0269
Intrinsic Subtyping: HER2+ vs Basal	2.50472 (0.7916–7.9253)	0.1182	2.76312 (0.85292–8.9515)	0.0901
Intrinsic Subtyping: Luminal-A vs Basal	0.94615 (0.2521–3.5514)	0.9346	1.05112 (0.27204–4.0614)	0.9424
Intrinsic Subtyping: Luminal-B vs Basal	1.98719 (0.5674–6.9599)	0.2829	2.19388 (0.60928–7.8997)	0.2294
Intrinsic Subtyping: Normal vs Basal	1.29351 (0.4278–3.9114)	0.6485	1.38074 (0.44619–4.2727)	0.5756
Recurrence Score: Intermediate vs High	0.74021 (0.4402–1.2447)	0.2566	0.75085 (0.44564–1.2651)	0.2817
Recurrence Score: Low vs High	0.69454 (0.4449–1.0842)	0.1087	0.73289 (0.46809–1.1475)	0.1743
Mammaprint: Poor vs Good	1.48393 (0.9134–2.4109)	0.1109	1.39329 (0.85261–2.2769)	0.1856
76-Gene Signature: Poor vs Good	1.31577 (0.8733–1.9825)	0.1895	0.77205 (0.52729–1.1304)	0.1925
Sotiriou: Luminal-like vs Basal-like	0.80253 (0.5487–1.1737)	0.2567	0.53301 (0.23901–1.1886)	0.1836
Mira: Poor vs Good	0.53318 (0.2391–1.189)	0.1243	0.46635 (0.24924–0.8726)	0.1241
BPMS: BPMS- vs BPMS+			0.4878 (0.261–0.912)	0.017
**Table 3B**				
Model	Log-likelihood			
S∼prolif + pam50 + RS + mamma + 76gene + sot + mira	−713.11			
S∼prolif + pam50 + RS + mamma + 76gene + sot + mira + BPMS	−710.71			
−2*(L0 – L1)	4.8			
	p-value = 0.028			

**Multivariate cox models for prognostic signatures**. Survival data was fit in the BrCa443 validation set against the 7 combined signatures in a multivariate Cox proportional hazards model. Similarly, the same data was fit in the BrCa443 set against the 7 combined signatures including the BPMS. B) **Likelihood ratio test for competitive models**. Using the likelihood of the multivariate models, an analysis of variance (likelihood ratio test) demonstrates that the BPMS selects a cohort of patients independent of all other gene signatures (p = 0.028, χ^2^ = 4.8, df = 1). A)

### BPMS signature is effective in other array platforms

To test for the potential of cross platform use, we applied the BPMS to the recently-derived METABRIC expression dataset generated from 2000 heterogeneous breast cancer tumors using Illlumina BeadArrays. Using the BPMS, we observed a more modest but significant stratification of high risk METABRIC patients (p-value = 0.0481; Figure S4). Thus, the BPMS has utility even when applied to different platforms.

## Discussion

New tools to classify TNBCs are urgently required in order to improve our prognostic capability and predict response to therapy. Here we utilized a novel optimization strategy to define a gene expression signature, BACH1 Pathway Metastasis Signature (BPMS), that significantly and selectively stratifies metastasis-free survival (MFS) in basal-like and, in particular, highest risk TNBC patients. This signature stratifies high risk patients within the TNBC cohort using only one-third the number of genes of our previously published RKIP-based pathway signature. The BPMS also further stratifies patients with apparent good prognosis as assessed by multiple molecular signatures as well as identifying patients at highest risk among those clinically classified as having poor prognoses using the Mammaprint® and Oncotype® signatures. Finally, the BPMS has potential clinical application as a single sample predictor.

The BPMS was derived from a previously defined RKIP Pathway Metastasis Signature (RPMS) that identifies a cohort with significantly poorer prognosis than patients outside the cohort. The RPMS achieves patient selection by a process that utilizes the expression levels of 7 gene products, which we defined as the RPMS target gene set: *RKIP*, meta-let-7-TG, meta-BACH1, *HMGA2*, *MMP1*, *CXCR4*, and *OPN*. By contrast, the BPMS described here differs from the RPMS on several levels. First, the *BACH1* meta-gene was based on experimentally defined gene targets in the BPMS rather than predicted targets as in the RPMS. Second, the original RPMS signature utilized approximately 100 genes whereas this number was reduced to 30 in the BPMS. To achieve this, we simply selected for gene targets with lowest overall variance. Third, to set the threshold for patient selection for the BPMS, we used an optimization approach that utilizes machine learning rather than the median threshold that we used to generate the RPMS. To assess the significance of our 7 selected genes, we analyzed random gene sets in comparison to the genes in the BPMS. We demonstrated that our signature using the BPMS target gene list performs better than 95% of random 7-gene signatures produced using our optimization and cross-validation methodology. Together, our results demonstrate that the BPMS is selective and significant as an analytical tool for patient outcome related to MFS.

The BPMS has potential clinical relevance in that it significantly enhances the ability of single tests to predict future prognosis for patients. Surprisingly, analysis of four recent molecular signatures using the BPMS shows that some patients classified as low risk were in fact high risk. With the advent of targeted therapy, it is possible that some of these signatures may be applied in a clinical setting. For example, beta blocker treatment is currently being evaluated for TNBC patients that could be stratified by a glucocorticoid receptor signature [27]. However, our results suggest that a beta blocker alone will not be sufficient to treat the patients with the poorest prognosis. In such cases, additional application of the BPMS would have direct clinical impact by identifying and removing this highest risk patient group from treatment.

The potential clinical impact of the BPMS relates primarily to targeted therapy. Among high risk patients, further stratification of the patients at highest risk would have limited clinical impact if standard therapeutic treatment is used for all patients with a poor prognosis. Currently, effective targeted therapy is lacking for patients with TNBC. However, further stratification using the BPMS enables identification of the most at-risk subgroup for the development of targeted therapies directed at this subgroup, a goal of personalized medicine.

The BPMS has the potential to identify therapeutic targets for some of the most invasive TNBC patients. Technically, the genes in the signature are biomarkers for a specific signaling environment within tumor cells. However, we developed the signature through a combination of experimental and clinical validation of the key driver genes in the signature [13]. Thus the signature is based upon a mechanistic signaling relationship between genes that could potentially be disrupted to obtain a more favorable outcome. Our previous studies have shown that genes forming the basis of the BPMS such as *BACH1* are promoters of metastasis and would be important therapeutic targets [14]. For example, *BACH1* is negatively regulated by hemin [28], an FDA-approved drug used to treat porphyria (Panhematin®, Lundbeck Inc, Deerfield, IL). Since BACH1 regulates antioxidants [29], there are likely to be other potential FDA-approved therapies for targets downstream of *BACH1* as well [30], [31]. In contrast to gene expression-based clustering and classification, the focus on molecular targets that drive cellular signaling pathways in tumor cells in combination with the ability to use the BPMS to select these particular patient populations has high potential as a future therapeutic strategy.

The goal of personalized medicine is to provide a single patient with detailed information that uniquely categorizes that individual, indicating a personalized course of treatment. To that end, the BPMS can be used to identify a high risk cohort of patients in which our signaling pathway is driving metastatic events. With this information, it may be possible to provide targeted therapy for individuals classified as BPMS-positive using our knowledge of the signaling pathway. Further application of our methodology may also be used to identify different signaling pathways that drive similar metastatic events in breast or other tumor types.

## Supporting Information

Figure S1 The Let-7-TG and BACH1 meta-genes exhibit a normal distribution of expression in breast tumors. Q-Q plots were used to verify the normal distribution of (A) Let-7-TG and (B) BACH1 meta-genes. Meta-gene values were analyzed using the BrCa871 dataset. The red line refers to an idealized normal distribution of gene expression.
(EPS)

Figure S2 The BPMS in GR-/ER-. BPMS within the cohort of patients classified as GR-/ER-. GR- patients were classified using GR probe expression below the 25th quartile. Similarly, ER- patients were classified using ESR1 probe expression below −3.416.
(EPS)

Figure S3 The BPMS is limited as a prognostic signature for low risk patients. Clinically relevant gene signatures (A) Mammaprint® Good, (B) OncotypeDX® Recurrence Low, or (C) OncotypeDX® Recurrence Intermediate were stratified for MFS using the BPMS. Red indicates patient tumors that express the BPMS signature while black indicates patient tumors that do not. Survival curves were generated by Kaplan–Meier analysis, and the indicated P-values were calculated by the log-rank test.
(EPS)

Figure S4 The BPMS is prognostic for metastasis-free survival (MFS) of patients in the METABRIC cohort. The METABRIC expression data set was generated from 2000 heterogeneous breast cancer tumors using Illlumina BeadArrays. Red indicates patient tumors that express the BPMS signature while black indicates patient tumors that do not. Survival curves were generated by Kaplan–Meier analysis, and the indicated P-values were calculated by the log-rank test.
(EPS)

Table S1 All genes differentially expressed (p<0.001) from BACH1 depletion in a TNBC cell line. Gene up-regulated (left three columns) and down-regulated (right three columns) through stable expression of shBACH1 when compared to vector control in MDA-MB-231 derived 1833 cells.
(DOCX)

Methods S1 Supplemental information regarding data pre-processing methodology, meta-gene construction, multivariate survival analysis, generation of the signature, and methodology regarding signature comparisons.
(DOCX)
