# Supplementary material for: A Prognostic Gene Signature for Metastasis-Free Survival of Triple Negative Breast Cancer Patients
Source: PLoS One. 2013 Dec 11;8(12):e82125. doi: 10.1371/journal.pone.0082125 (PMC3859562; doi:10.1371/journal.pone.0082125)
Supplement: Table S1 — All genes differentially expressed (p<0.001) from BACH1 depletion in a TNBC cell line. Gene up-regulated (left three columns) and down-regulated (right three columns) through stable expression of shBACH1 when compared to vector control in MDA-MB-231 derived 1833 cells. (DOCX) [file pone.0082125.s005.docx]

**Table S1. All genes differentially expressed (p < 0.001) from BACH1 depletion in a TNBC cell line.** Gene up-regulated (left three columns) and down-regulated (right three columns) through stable expression of shBACH1 when compared to vector control in MDA-MB-231 derived 1833 cells.

| **Symbols** | **p-values** | **Fold Change** | **Symbols** | **p-values** | **Fold Change** |
| --- | --- | --- | --- | --- | --- |
| **PLCB4** | **3.36E-05** | **1.60** | **MAML2** | **1.10E-03** | **0.93** |
| **CDH18** | **3.84E-05** | **1.42** | **NARS** | **1.28E-03** | **0.93** |
| **TULP3** | **3.60E-05** | **1.38** | **TTC30B** | **1.40E-03** | **0.93** |
| **TMPRSS15** | **1.26E-04** | **1.36** | **IFI27** | **1.52E-04** | **0.92** |
| **NUP210** | **5.29E-05** | **1.33** | **AMPH** | **4.27E-04** | **0.92** |
| **BST2** | **1.06E-03** | **1.31** | **CD177** | **1.37E-03** | **0.92** |
| **GABBR2** | **2.42E-04** | **1.30** | **ALG10B** | **7.37E-04** | **0.92** |
| **SLC1A3** | **1.47E-04** | **1.29** | **RNF19B** | **1.02E-03** | **0.92** |
| **LTV1** | **4.56E-05** | **1.28** | **NIPAL3** | **7.48E-04** | **0.91** |
| **WNT7B** | **3.12E-05** | **1.28** | **FHOD3** | **8.17E-04** | **0.91** |
| **KCNAB2** | **2.44E-04** | **1.28** | **HS3ST3B1** | **1.30E-03** | **0.91** |
| **PEX3** | **7.30E-05** | **1.24** | **PTGR1** | **1.44E-03** | **0.91** |
| **GFPT2** | **1.87E-04** | **1.24** | **FAM160B1** | **1.41E-03** | **0.91** |
| **F13A1** | **1.30E-04** | **1.22** | **RIOK3** | **8.53E-04** | **0.91** |
| **PIK3CG** | **6.46E-04** | **1.22** | **DDX60L** | **7.22E-04** | **0.91** |
| **FBXO30** | **5.05E-05** | **1.21** | **HERC3** | **1.26E-03** | **0.91** |
| **KIAA1467** | **1.02E-04** | **1.21** | **MEX3C** | **1.38E-03** | **0.90** |
| **ADD2** | **4.19E-04** | **1.21** | **C5orf30** | **8.76E-04** | **0.90** |
| **AIG1** | **8.38E-05** | **1.21** | **RCAN3** | **3.99E-04** | **0.90** |
| **FUCA2** | **2.84E-04** | **1.20** | **EMB** | **1.99E-04** | **0.90** |
| **SF3B5** | **3.59E-04** | **1.20** | **GSPT2** | **2.50E-04** | **0.90** |
| **RAB15** | **1.92E-04** | **1.20** | **ADAMTS1** | **1.47E-03** | **0.90** |
| **PHACTR2** | **1.77E-04** | **1.20** | **FNDC3A** | **1.17E-03** | **0.90** |
| **RAP1GAP2** | **1.35E-03** | **1.19** | **ZSCAN30** | **3.89E-04** | **0.90** |
| **EYA4** | **8.14E-05** | **1.19** | **C18orf54** | **4.58E-04** | **0.90** |
| **SCARA3** | **1.95E-04** | **1.19** | **IL7** | **1.64E-04** | **0.90** |
| **C3** | **1.10E-03** | **1.19** | **KIAA0495** | **5.25E-04** | **0.90** |
| **PLD5** | **6.96E-04** | **1.18** | **SGMS1** | **6.48E-04** | **0.89** |
| **C6orf192** | **3.19E-04** | **1.18** | **RAD50** | **1.14E-03** | **0.89** |
| **ADAT2** | **1.22E-03** | **1.18** | **PIAS2** | **8.07E-04** | **0.89** |
| **EEF1A2** | **5.71E-04** | **1.17** | **OSBPL6** | **6.82E-04** | **0.89** |
| **AHI1** | **1.20E-04** | **1.17** | **LRRN1** | **6.79E-04** | **0.89** |
| **GPR17** | **1.46E-03** | **1.17** | **ARPP19** | **4.41E-04** | **0.89** |
| **NPEPPS** | **4.47E-04** | **1.16** | **TBXAS1** | **3.81E-04** | **0.88** |
| **PLXNA1** | **9.14E-05** | **1.16** | **SMAD4** | **2.36E-04** | **0.88** |
| **MYBL2** | **3.64E-04** | **1.16** | **KAL1** | **1.43E-03** | **0.88** |
| **TBPL1** | **2.19E-04** | **1.15** | **MAPK13** | **9.75E-04** | **0.88** |
| **HBS1L** | **4.99E-04** | **1.15** | **STXBP2** | **3.61E-04** | **0.88** |
| **INHBB** | **1.69E-04** | **1.15** | **TNFRSF11B** | **1.36E-04** | **0.88** |
| **COX7C** | **3.30E-04** | **1.15** | **COBLL1** | **1.24E-03** | **0.88** |
| **PCSK6** | **3.51E-04** | **1.14** | **PLAT** | **4.80E-04** | **0.88** |
| **ATXN7L3B** | **1.50E-04** | **1.14** | **NA** | **4.67E-04** | **0.87** |
| **AGAP2** | **4.69E-04** | **1.14** | **TLR4** | **3.49E-04** | **0.87** |
| **GCLM** | **1.38E-03** | **1.14** | **EFEMP1** | **6.29E-05** | **0.87** |
| **STX11** | **2.52E-04** | **1.14** | **ATP8B1** | **5.82E-04** | **0.87** |
| **CCDC28A** | **8.69E-04** | **1.14** | **WASF3** | **9.02E-04** | **0.87** |
| **SHANK1** | **4.30E-04** | **1.13** | **C16orf62** | **2.93E-04** | **0.87** |
| **DMD** | **1.28E-04** | **1.13** | **TGFBI** | **6.62E-04** | **0.87** |
| **C9orf86** | **3.54E-04** | **1.12** | **DYM** | **1.15E-04** | **0.87** |
| **SFXN2** | **8.02E-04** | **1.12** | **LRP1** | **6.50E-04** | **0.87** |
| **GPR126** | **8.22E-04** | **1.12** | **AKD1** | **7.06E-05** | **0.87** |
| **CMTM3** | **1.23E-03** | **1.12** | **LACE1** | **9.69E-05** | **0.86** |
| **ZAK** | **7.28E-04** | **1.12** | **BMPER** | **1.40E-03** | **0.86** |
| **SCARB1** | **1.02E-03** | **1.12** | **SKIL** | **3.86E-04** | **0.86** |
| **KLF11** | **1.03E-03** | **1.12** | **PELI1** | **1.47E-03** | **0.86** |
| **HMOX1** | **9.10E-04** | **1.12** | **RFPL4A** | **1.33E-03** | **0.86** |
| **VTA1** | **8.15E-04** | **1.11** | **GALNT14** | **7.69E-04** | **0.86** |
| **LSP1** | **1.04E-03** | **1.11** | **RASGRP3** | **1.11E-03** | **0.86** |
| **DMKN** | **1.46E-03** | **1.11** | **MGAT4A** | **8.30E-04** | **0.85** |
| **PERP** | **5.80E-04** | **1.11** | **CCDC80** | **1.34E-03** | **0.85** |
| **AVPI1** | **1.48E-03** | **1.11** | **SELENBP1** | **1.13E-04** | **0.85** |
| **CYB5A** | **8.13E-04** | **1.10** | **PRDM1** | **2.22E-04** | **0.85** |
| **AK4** | **8.39E-04** | **1.10** | **GPR116** | **2.67E-04** | **0.84** |
| **MED23** | **1.42E-03** | **1.10** | **EPB41L4A** | **1.18E-03** | **0.84** |
| **GLB1L2** | **5.52E-04** | **1.10** | **MAGEC1** | **6.17E-04** | **0.84** |
| **REPS1** | **3.14E-04** | **1.10** | **PRR16** | **5.57E-04** | **0.84** |
| **EIF4E2** | **1.10E-03** | **1.10** | **TIE1** | **1.97E-04** | **0.83** |
| **PLCD3** | **2.17E-04** | **1.09** | **ANO5** | **8.05E-04** | **0.82** |
| **ZMAT4** | **9.41E-04** | **1.09** | **CYP24A1** | **8.72E-04** | **0.82** |
| **PCDHB3** | **8.25E-04** | **1.09** | **FAM83A** | **5.77E-05** | **0.81** |
| **MTHFSD** | **5.69E-04** | **1.08** | **CHRM3** | **1.19E-03** | **0.81** |
| **DKFZp686O24166** | **7.40E-04** | **1.08** | **LOX** | **2.79E-04** | **0.80** |
| **RHOB** | **1.40E-03** | **1.08** | **1-Mar** | **4.08E-04** | **0.80** |
| **HS6ST1** | **1.31E-03** | **1.08** | **FIG4** | **3.02E-04** | **0.80** |
| **MRPL39** | **1.37E-03** | **1.08** | **ENTPD3** | **1.18E-04** | **0.78** |
| **EPHB4** | **3.97E-04** | **1.07** | **SAMD12** | **6.53E-05** | **0.78** |
| **C3orf23** | **1.22E-03** | **1.06** | **FRMPD4** | **4.81E-05** | **0.77** |
| **LOC729444** | **6.15E-04** | **1.06** | **PDE1C** | **8.62E-05** | **0.77** |
| **CTDP1** | **1.32E-03** | **1.06** | **TMEM45B** | **7.54E-05** | **0.72** |
|  |  |  | **PLEKHA7** | **6.26E-04** | **0.72** |
|  |  |  | **SPOCK1** | **4.08E-05** | **0.72** |
|  |  |  | **NAP1L3** | **1.38E-04** | **0.70** |
|  |  |  | **EHF** | **6.29E-04** | **0.69** |
|  |  |  | **MYCT1** | **4.32E-05** | **0.69** |
|  |  |  | **TSPYL5** | **7.63E-04** | **0.68** |
|  |  |  | **HIST1H2BA** | **1.79E-04** | **0.54** |
|  |  |  | **UCA1** | **5.53E-05** | **0.50** |
