# Supplementary material for: A Prognostic Gene Signature for Metastasis-Free Survival of Triple Negative Breast Cancer Patients
Source: PLoS One. 2013 Dec 11;8(12):e82125. doi: 10.1371/journal.pone.0082125 (PMC3859562; doi:10.1371/journal.pone.0082125)
Supplement: Methods S1 — Supplemental information regarding data pre-processing methodology, meta-gene construction, multivariate survival analysis, generation of the signature, and methodology regarding signature comparisons. (DOCX) [file pone.0082125.s006.docx]

**SUPPLEMENTAL METHODS**

**Data set pre-processing**

Three data sets were assembled from 11 cohorts retrieved from the Gene Expression Omnibus (GEO) based on previously published studies. All gene expression arrays were conducted on the Affymetrix Human Genome u133a platform, with the exception of the METABRIC data set, which was performed on Illumina Bead Arrays. The transcripts were isolated from breast cancer tumors that were annotated with clinical follow up including metastasis-free survival data. The training set BrCa871, consisting of 871 patients, contains five cohorts identified by their GEO accession numbers: GSE1456, GSE2990, GSE3494, GSE7390, and GSE11121. The first testing data set BrCa443 (443 patients) is composed of three cohorts: GSE5327, GSE2034, and GSE2603 and the second testing set BrCa341 (341 patients) is also composed of three cohorts: GSE6532, GSE12093, GSE31519.

Cohorts were extracted as compressed raw CEL files into a single directory for each BrCa871, BrCa443, and BrCa341 data set. The CEL files were then converted into Biobase (R library ‘Biobase’, v 2.16.0) ExpressionSet objects in R (versions 2.13.0 through 2.15.0) using the default R Affymetrix package (R library ‘affy’0, v 1.34). Data sets were then RMA preprocessed (R library ‘affy’). If multiple probes mapped to a single gene in the HG-u133a package (R library ‘hgu133a.db’), the probe with the highest overall variance was selected. Each array was subsequently normalized ~ N(0,1) by sample in BrCa871, BrCa443, and BrCa341 and by gene in the METABRIC data set. Each sample was also median-centered.

By definition, batch effects are data trends that correspond to non-biological effects. One method that has been used to account for data-processing batch effects is data normalization which we also performed. However, as all of our cohorts have biological differences, trends apparent in the publicly available data relating to data source cannot be assumed to be non-biological [[1](#_ENREF_1)].

**Meta-gene construction**

In order to define a measure of *let-7* and BACH1 activity we generated their corresponding meta-genes, metaLET*7* and metaBACH1 respectively, each defined as a weighted average of the transcriptional levels of their downstream targets. The relative weights were individual gene scores calculated in the Gene Set Analysis package (R library ‘GSA’), and served as an estimate of regulatory strength of the given regulator (*let-7* or BACH1) to its target genes.

In preprocessing, where multiple probes can map to single genes, we selected those probes with highest overall variance to represent expression. We sought to minimize the number of genes by selecting a smaller target set for both metaLET7 and metaBACH1 in a fashion that would maximize consistency across data sets. An initial list of targets was constructed for the metaLET7 target gene group using the TRANSFAC database while a similar list was constructed by applying 'samr' to shBACH1 1833 cells. Beginning with these initial genes, we selected for gene targets with lowest overall variance in the BrCa871 data set. For the *let*-7 target gene set, the 12 targets with lowest variance were selected, excluding BACH1 and *HMGA2* as they are represented separately in the signature. Similarly, we chose the 13 targets of BACH1 with lowest variance to represent the BACH1 target gene set.

**Multivariate survival analysis**

All survival analysis was performed using the R library 'survival'. As the BPMS is a binary classifier (patients are either BPMS-positive or BPMS-negative), the log-rank test of significance was used (implementation as R commands 'survdiff' or 'coxph', library 'survival'). Comparison between existing prognostic signatures was performed using linear Cox proportional hazards models. Further comparison against prognostic signatures was performed by applying the log-rank test for the BPMS within individual cohorts of those signatures.

Linear Cox proportional hazards models for metastasis-free survival were fit against a large range of prognostic breast cancer signatures (R function 'coxph', library 'survival'). Two sets of gene signatures were employed: a primary set and a secondary set. Full Cox models were fit using the entirety of the primary set as well as a set consisting of both the primary and secondary sets. For each of these two models, additional models were fit including the BPMS. In total, there are four models: A) Surv(MFS, met) ~ primary set, B) Surv(MFS, met) ~ primary set + BPMS, C) Surv(MFS, met) primary set + secondary set, D) Surv(MFS, met) ~ primary set + secondary set + BPMS. Using the likelihood of each of these models, we can test the hypothesis that the model with the BPMS is more likely to explain the data than the model not including the BPMS. This is called the likelihood-ratio test. To be specific, models B and A were compared, as were models D and C. This results of the analysis are reported in the main text. A summary of this test as well as hazard ratios with 95% confidence intervals for these fits can be found in Supplemental Table 3.

While the likelihood-ratio test can evaluate whether the addition of BPMS against an aggregation of other gene signatures is significant, examining the stratifications of the BPMS within individual subgroups determined by other prognostic signatures (such as the BPMS within the Basal cohort of the PAM50) allows for an more specific analysis of the intersection of these signatures.

**Generation of the BPMS**

In the provided R code, the function 'genBPMSSig' can be used to generate estimates for the thresholds in the BPMS signature. The 'genBPMSSig' function generates a distribution of thresholds that will produce significance in a training set and a validation set – therefore the quality of the estimate depends on the size of the distribution. As such, 'genBPMSSig' takes as parameters a data set for training, a data set for cross-validation, the set of BPMS genes, and n number of potential solutions that the function will generate to create the final estimates.

A number of solutions (n) is generated within the 'genBPMSSig' function through a call to the function 'analysisPipelineRPMS.v2'. This pipeline function optimizes for each n solutions a cost function as described in the main text. This cost function is the function 'ensembleCostFcn.v2' and can be found in the provided R code. Optimizations were performed using the downhill simplex algorithm as implemented in the R function 'optim' and described in the main text.

Given these n potential solutions, the significance of these solutions is calculated in both the training and cross-validation sets. Those solutions producing significance in both sets were then averaged to form a final set of BPMS thresholds. A summary of this process is provided in Figure 2 as a flow-chart.

**Comparison against random signatures**

Following the pipeline described in the previous section, the function 'genPValueRandomGenes' applies the same optimization and estimation process to m number of random 7-gene signatures using an identical model and cost function. This process produces a distribution of p-values for random 7-gene signatures. Statistically, this distribution of p-values should follow a uniform distribution. Using this distribution of p-values, the probability of producing the BPMS signature as a random effect is less than 5%, as described in the manuscript.

**REFERENCES**

1. Leek JT, Scharpf RB, Bravo HC, Simcha D, Langmead B, et al. (2010) Tackling the widespread and critical impact of batch effects in high-throughput data. Nat Rev Genet 11: 733-739.
